# Use of the Internet and Digital Devices Among People With Severe Mental Ill Health During the COVID-19 Pandemic Restrictions

**DOI:** 10.3389/fpsyt.2021.732735

**Published:** 2021-09-24

**Authors:** Panagiotis Spanakis, Paul Heron, Lauren Walker, Suzanne Crosland, Ruth Wadman, Elizabeth Newbronner, Gordon Johnston, Simon Gilbody, Emily Peckham

**Affiliations:** ^1^Mental Health and Addiction Research Group, Department of Health Sciences, University of York, York, United Kingdom; ^2^Independent Peer Researcher, Clackmannan, United Kingdom

**Keywords:** severe mental ill health, psychosis, bipolar, internet, digital devices, digital divide, COVID-19

## Abstract

**Background:** Restrictions due to the COVID-19 pandemic have led to everyday reliance on digitalisation of life, including access to health care services. People with severe mental ill health (SMI—e.g., bipolar or psychosis spectrum disorders) are at greater risk for digital exclusion and it is unknown to what extent they adapted to online service delivery. This study explored use of the Internet and digital devices during the pandemic restrictions and its association with physical and mental health changes.

**Methods:** Three hundred sixty seven adults with an SMI diagnosis completed a survey (online or offline) and provided information on access to Internet connexion and devices, internet knowledge, online activities, and barriers to using the Internet. They also self-reported changes in mental and physical health since the beginning of the pandemic restrictions.

**Results:** During the pandemic restrictions 61.6% were limited or non-users of the Internet. The majority had access to the Internet and digital devices but around half reported knowledge deficits. Most common activities were accessing information and entertainment (88.9%), staying in touch with friends and families (84.8%), and purchasing goods (other than food) (84.3%). Most common barriers were finding the Internet “not interesting” (28.3%) or “too difficult” (27.9%), as well as “security concerns” (22.1–24.3%). Using the Internet “a lot” (vs. “just a bit or not at all”) during the pandemic was associated with younger age (18–30: Adj ORs 4.76; 31–45: 6.39; *Ps* < 0.001; vs. 66+), having a diagnosis of bipolar disorder (compared to psychosis; Adj OR = 3.88, *P* < 0.001), or reporting a decline in mental health (compared to no decline; Adj OR = 1.92, *P* = 0.01).

**Conclusion:** Most people with SMI were limited or non-users of the Internet during the pandemic, which seems to be mainly attributable to lack of interest and skills, rather than lack of devices or connectivity. Older adults with psychosis should be the focus of interventions to support digital engagement in people with SMI.

## Introduction

The COVID-19 pandemic led governments in many countries, including the UK, to impose restrictions in movement and social contact, to reduce the spread of the virus (1, 2). With travelling and face-to-face activities severely disrupted, people became more reliant on the Internet to perform daily activities such as keeping in touch with loved ones and accessing support (e.g., health services and purchasing essentials) (3, 4). In the UK's National Health Service (NHS), the pandemic restrictions led to a shift from traditional face to face care to remote (telephone or video call) care, both in mental health (5–7) and the broader sector (8–10).

However, sizeable sections of the UK society are either non-users [13% (11)] or only limited users of the Internet [using the Internet infrequently and for a small range of activities; 14.3%, (12)], with the main barriers being lack of access to the Internet and digital devices, lack of skills, or lack of motivation (3). During the pandemic, this might lead to digital exclusion via restricted or no access to online services and activities. For example, during the early phases of the pandemic, people who were considered at risk for severe complications from COVID-19 were sent a letter containing multiple web links to sources of support. However, it was estimated that around 150,000–175,000 people sent this letter did not have access to the Internet (13).

Worryingly, vulnerable groups such as older adults, and disabled or displaced people, are more likely to be digitally excluded (14, 15). One such group that has traditionally faced profound inequalities are people with severe mental ill health (SMI) such as psychosis spectrum and bipolar disorders; despite this they have received very little attention in terms of digital exclusion risk. People with SMI are likely to need to attend regular health care appointments to monitor their health conditions. Often, they suffer from long-term physical illnesses leading to reduced life expectancy (16, 17) and, therefore, they have been more likely to self-isolate for long periods of time during the pandemic. The pandemic restrictions were also likely to exacerbate feelings of loneliness that were already common among people with SMI prior to the pandemic (18). In this context, use of the Internet might have been vital for people with SMI to access health care and support for their physical and mental health needs, as well as informal social support and information during the pandemic restrictions. Pre-pandemic data suggest that digitally engaged people with SMI found digital technology helpful for coping with their illness, as for example using music to manage auditory hallucinations, finding mental health information online, and monitoring symptoms and medication (19).

Pre-COVID data shows a mixed picture of digital engagement in people with SMI. In a sample of people with bipolar disorder almost everyone owned a smartphone or computer (20). Among people with psychosis-spectrum disorders the majority owned a computer and about half had access to a smartphone (19), although lower rates were reported in a meta-analysis of smartphone ownership (about a third had a smartphone) (21). However, some of these findings (19, 20) are based on exclusively online surveys, and we do not know if they would generalise to non-users of the Internet. Furthermore, a meta-analysis demonstrated favourable attitudes toward mHealth services (e.g., using mobile phones to monitor symptoms and receive health information) (21), while a trial about videoconferencing care services found that although service users expressed high rates of satisfaction, actual uptake of the service was quite low (22). A longitudinal study of people with psychosis found that although digital exclusion reduced from 30 to 18.3% over a 5 year period, it still affected a large minority of people with SMI (23). In that study authors also noted that a wide divide still existed at that time between people with SMI and the general population in daily internet use (56% in SMI vs. 78% in the general population). Other findings have also suggested that rates of digital exclusion were much higher in people using community rehabilitation services and thus more profoundly affected by their SMI condition [only 14.4% were Internet users; (24)].

Although some of these findings are encouraging, there is need for updated research, considering the vast digitalisation of services that happened during the pandemic, including people across a range of diagnosis (e.g., both psychosis-spectrum and bipolar disorders) and digital engagement levels (e.g., online as well as offline data collection methods). This is important to understand whether people with SMI have adapted to the increased digitalisation of life and remain connected to their sources of support. To address this knowledge gap, the Optimising Well-being in Self Isolation (OWLS) survey explored the digital experiences of people with SMI during the pandemic restrictions. The aim was to identify the extent to which people with SMI have been using the Internet, whether socio-demographic and health characteristics had any influence on this, and whether Internet use was associated with changes in mental or physical health. We also sought to understand what people have been using the Internet for and what barriers exist to this.

## Methods

### Design and Procedure

This study reports on results from the Optimising Well-being in Self-isolation (OWLS) study, which recruited participants from the Closing the Gap (CtG) study. The CtG study was a large clinical cohort (*N* = 9,914) of people with severe mental ill health, recruited between April 2016 and May 2020 (25) from areas across England apart from the East of England and West Midlands. The inclusion criteria where being 18 years or older and having a documented diagnosis of schizophrenia or delusional/psychotic illness (ICD 10 F20.X & F22.X or DSM equivalent) or bipolar disorder (ICD F31.X or DSM equivalent). OWLS recruited a sub-cohort of CtG from July 2020 to December 2020, to explore the effects of the COVID-19 pandemic restrictions on people with severe mental ill health (26).

Selection of participants from the CtG study to be invited to OWLS followed a stepwise process. First, researchers identified all CtG participants that have consented to be contacted again, have provided their contact details, and have been originally recruited from a clinical site that had the capacity to collaborate with us in a new research project. Eligible participants were then organised in groups based on age, gender, ethnicity, and care setting (primary or secondary mental health care) to ensure representation across many socio-demographic groups. From each group, researchers selected a purposive sample of participants that had most recently participated in the CtG study (e.g., recruited in the last 2 years) ensuring that a range of localities was covered. Recent participation to the CtG was considered important to increase response rates (e.g., the team having current and valid contact details, and participants being familiar with the research team). Locality was used to provide geographical diversity, inviting participants from 17 mental health trusts and six Clinical Research Network (CRN) areas in England, including a mix of rural and urban settings. Participants were recruited from North England, East Midlands/Anglia, London, and South England.

Participants were invited by phone, text, or email and provided with an information sheet (read over the phone, or send by email, text message, or post). Those consenting to participate were given the option to complete the survey over the phone with a researcher, online, or completing and returning a hard copy survey sent by post.

Ethical approval was granted by the Health Research Authority Northwest—Liverpool Central Research Ethics Committee (REC reference 20/NW/0276).

### Measures

All the measures and analyses presented here are pre-registered (27) in Open Science Framework (OSF). As the registered plan refers to the entire OWLS project, readers may consult section sample characteristics in analysis, as well as the sub-sections denoted in this paper.

#### Sample Characteristics

In the CtG study, participants provided information on their date of birth, ethnicity, and areas of residence. Date of birth was used to calculate participants' age at the time they took part in OWLS and ethnicity was coded as White or Other than White. Participants post-codes were used to obtain a measure of socioeconomic deprivation based on the scores assigned by the Ministry of Housing, Communities and Local Government (28). Scores range from 1 to 10 with higher scores indicating lower deprivation. Participants were categorised as residing in areas of very high (1, 2), high (3, 4), medium (5, 6), low (7, 8), or very low deprivation (9, 10).

For participants that provided consent to obtain their SMI diagnosis details from their health records, diagnosis was categorised into psychosis (including schizophrenia, schizoaffective, or any other psychotic disorder), bipolar disorder, or other SMI (including 23 participants who took part in the CtG study based on an eligible diagnosis, but their diagnosis later changed to a different one, as for example depression with psychotic features). For 48 participants that did not provide consent or gave insufficient identifiable information (e.g., name and date of birth), diagnosis was coded as “not recorded.” Although the not-recorded category was not included in our registered plan, it was added to avoid dropping these 48 participants from the analysis.

In the OWLS study, participants who reported currently receiving support from mental health services were coded as secondary care patients, while those who were not receiving support from mental health services were coded as primary care patients. Participants also reported their financial situation since the beginning of the pandemic as “better,” “worse,” “about the same,” or “don't know.” After excluding those not knowing (*n* = 14, 3.8%) a binary variable was derived (decline or no decline in financial situation).

These measures are also presented in sections 4.2.2 and 4.5 in the pre-registered analysis plan (27).

#### Use of the Internet and Digital Technologies

Participants answered a series of single-item bespoke questions, created for the OWLS survey. These explored the following topics:

(a) Use of the Internet during the pandemic (In general, have you used the Internet during the pandemic restrictions to do some of your daily activities—e.g., buy groceries, pay bills, etc.?), with response options being: Yes a lot, Yes a little, or No. This was then categorised into users of the Internet (yes a lot) and limited/non-users of the Internet (yes a little, no) (See also section “Digital Engagement” in the registered plan).
(b) Participants who reported using the Internet (either a lot or a little) during the pandemic restrictions were asked to indicate the specific activities they performed, choosing as many as applied from a pre-specified list of activities.
(c) Access to digital devices (Do you own any of the following devices?), where participants could choose among smartphones, tablets, or laptop/desktop computers, and access to the Internet (Can you access the internet from your home—yes/no).
(d) Interest in learning about the Internet (Would you like to learn more about how to use the Internet to do some of your daily activities?), with response options being: There might be things I don't know and I would be interested in learning; there might be things I don't know but I am not interested in learning; and I already know how to do the things I want.

Self-reported knowledge of the Internet and barriers to using the Internet were assessed using items from the Oxford Internet Survey 2019 (available at https://oxis.oii.ox.ac.uk/). Self-reported knowledge was rated by participants from 0 (bad) to 4 (outstanding) and categorised as bad (0), poor/fair (1, 2), or good/outstanding (3, 4).

Participants who reported not using the Internet or using it “just a little” during the pandemic, were asked to indicate a reason for this, choosing as many as applied from a pre-specified list of barriers (e.g., I am just not interested, or it is too difficult to use). On advice of the OWLS Lived Experience Panel three of the original items from the Oxford Internet Survey were removed or adapted. For example, the item reading “it is not for people like me” was removed (language could have been perceived as stigmatising), and two items about data security concerns (I worry about being conned or having money stolen; I worry about having my personal details stolen) were rephrased into a single item using milder language (I worry about the security of my data and information) to reduce any potential triggers for psychotic symptoms (e.g., paranoia).

These measures are also reported in section *post-hoc* analysis in the registered plan.

#### Self-Reported Changes in Physical and Mental Health

Participants were asked how their subjective health has changed compared to before the pandemic restrictions, with the response options being: about the same; worse than before; better than before; I do not know. This was asked for physical and mental health separately. After removing those responding that they did not know (Physical Health: *n* = 11, 3.0%; Mental Health: *n* = 7, 1.9%), two separate binary variables were derived coded as decline in physical/mental health (including those reporting worse that before) or no decline (including all other options) [See also section 4.1 in the registered plan (27)].

#### Well-Being

Well-being was measured with the four items used by the Office of National Statistics (29), assessing life satisfaction, sense of worthwhileness, and feelings of happiness and anxiety. Each item was rated on a scale from 0 (not at all) to 10 (completely) and a total score (0–40) was calculated after reversing the last item. Higher scores indicated greater sense of well-being [See also section 4.1 in the registered plan (27)].

### Analysis

Descriptive statistics (Ns and frequencies) are presented for the sample characteristics and digital engagement variables described above. To understand who was more likely to use the Internet during the pandemic, we conducted a binary logistic regression that examined the association of age, ethnicity, socio-economic deprivation, treatment setting, and diagnosis with use of the Internet (user vs. limited/non-user). To understand the role of Internet use in people's health and well-being, we conducted a binary logistic regression that examined the association of well-being and changes in mental and physical health since the start of the pandemic with use of the Internet.

Associations of all independent variables and Internet use were examined with univariate models before added into the multivariate models. All independent variables were inserted into the multivariate model at once.

This paper also presents a *post-hoc* exploratory analysis to further investigate one of the findings derived from the registered analysis and its rationale is explained in the Results section. A chi-square test was used to examine the association of changes in mental health with age, as well as diagnosis. A binary logistic regression analysis was then conducted to examine the association of changes in mental health with use of the Internet, adjusting for age and diagnosis.

Analysis was conducted in IBM SPSS Statistics 26 and statistical significance was set at *P* < 0.05.

## Results

### Sample

**Figure 1:**
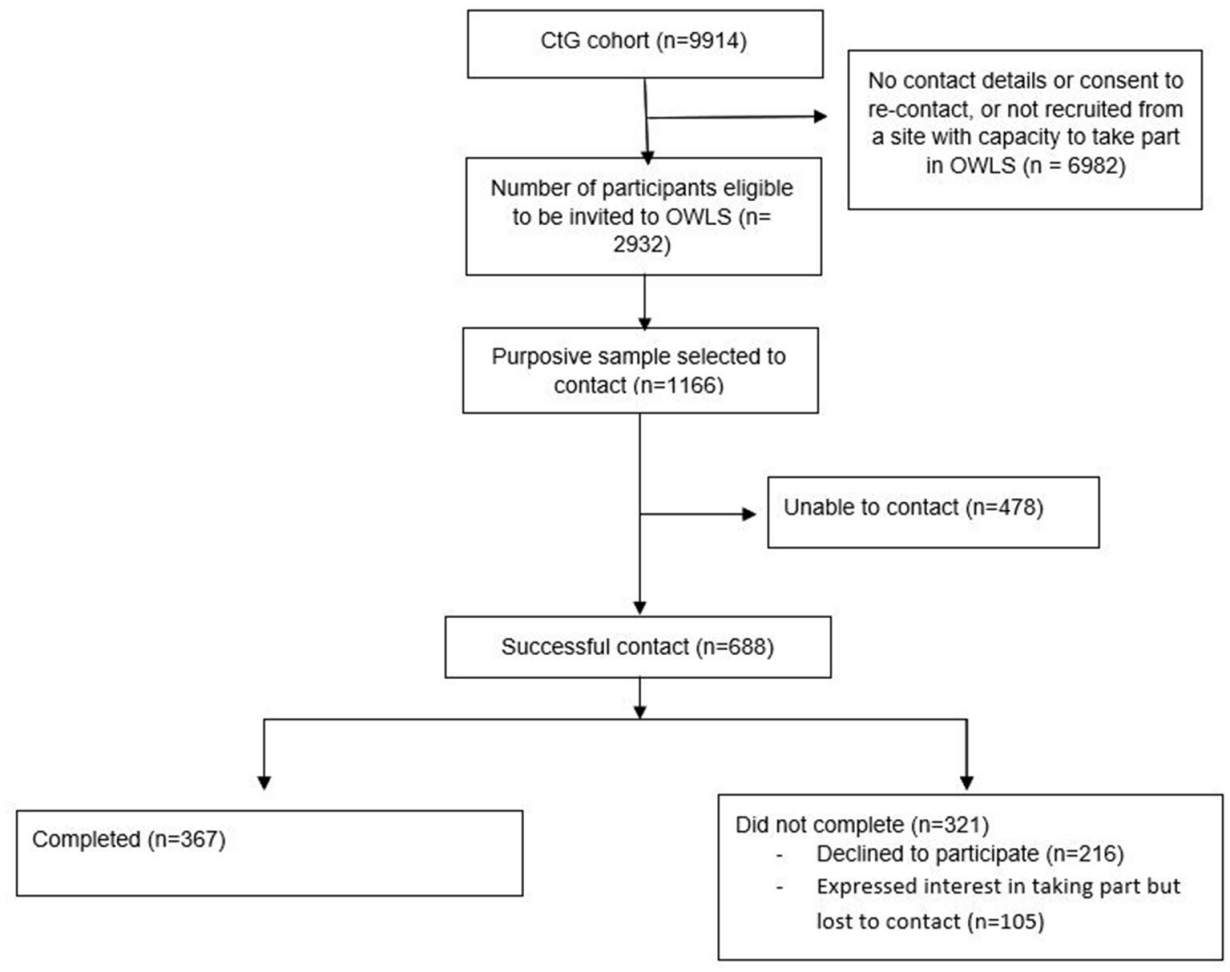
Flow diagram-OWLS.

Out of 2,932 participants in the CtG study that were eligible to be invited to OWLS, we selected a purposive sub-sample of 1,166 (39.8%) participants and successfully contacted 688 (59%). The survey was completed by 367 participants (31.5% of the selected sub-sample and 53.3% of those successfully contacted) (Figure 1).

The final study sample (*N* = 367) had a mean age of 50.5 (±15.69) years old and it included 51.0% men, 47.4% women, 1.6% transgender, 17.7% people from other than White ethnic background and 48.5% residing in high/very high deprivation areas in the country (Table 1). The primary diagnosis was psychosis (51.2%). The survey was completed online by 121 participants (33%) and over the phone or via the post by 246 (67%).

**Table 1:** Sample characteristics and health variables (*N* = 367).

**Variable**	***N*** **(%)^a^**
**Age**	
18–30	53 (14.4)
31–45	97 (26.4)
46–55	136 (37.1)
66+	81 (22.1)
Missing	0 (0.0)
**Gender**	
Male	187 (51.0)
Female	174 (47.4)
Transgender	6 (1.6)
Missing	0 (0.0)
**Ethnicity**	
Other than White	65 (17.7)
White	302 (82.3)
Missing	0 (0.0)
**Socioeconomic deprivation**	
Very high	97 (26.4)
High	81 (22.1)
Medium	67 (18.3)
Low	55 (15.0)
Very low	52 (14.2)
Missing	15 (4.1)
**Decline in financial situation during the pandemic**	
Yes	61 (16.6)
No	285 (77.7)
Don't know / Don't wish to answer	14 (3.8)
Missing (Included don't know)	7 (1.9)
**Mental health care setting**	
Primary care	139 (37.9)
Secondary care	224 (61.0)
Missing	4 (1.1)
**Diagnosis**	
Psychosis	188 (51.2)
Bipolar disorder	108 (29.4)
Other SMI	23 (6.3)
Not recorded	48 (13.1)
**Decline in mental health in the pandemic**	
Yes	148 (40.3)
No	210 (57.2)
Not sure/Don't know	7 (1.9)
Missing	2 (0.5)
**Decline in physical health in the pandemic**	
Yes	118 (32.2)
No	236 (64.3)
Not sure/Don't know	11 (3.0)
Missing	2 (0.5)

a *Percentages are out of total N = 367*.

### Digital Engagement Characteristics (Table 2)

During the pandemic restrictions, 136 participants (37.1%) were Internet-users, while 226 (61.6%) were limited or non-users of the Internet.

**Table 2:** Digital engagement characteristics (*N* = 367).

	***N*** **(%)^a^**
**Self-reported internet knowledge**	
Outstanding/good	179 (48.8)
Fair/Poor	129 (35.1)
Bad	39 (10.6)
Don't know	14 (3.8)
Missing	6 (1.6)
**Device ownership**	
Tablet/Smartphone	293 (79.8)
Laptop/Desktop	207 (56.4)
No device	49 (13.4)
Missing	0 (0.0)
**Internet access at home**	
Yes	308 (83.9)
No	54 (14.7)
Missing	5 (1.4)
**Would you like to learn more about the internet?**	
Already know what I need	178 (48.5)
There are things I do not know	182 (49.6)
I am interested in learning more	108 (59.3)^b^
I am not interested in learning more	74 (40.7)^b^
Missing	7 (1.9)
**Internet use during the pandemic**	
No	145 (39.5)
A little	81 (22.1)
No or a little (combined)	226 (61.6)
A lot	136 (37.1)
Missing	5 (1.4)
**Survey completion mode**	
Online	121 (33.0)
Phone/By post	246 (67.0)
Missing	0 (0.0)

a *Percentages are out of total N = 367*.

b *Percentages are out of N = 182 who identified a knowledge gap*.

Most of the participants owned a digital device and had access to the internet from home. Around half rated their knowledge of the Internet as good or outstanding and reported no knowledge gap (“*I already know what I need*”). Of those reporting a knowledge gap, 59.3% were interested in learning more about the internet (Table 2).

**Figure 2:**
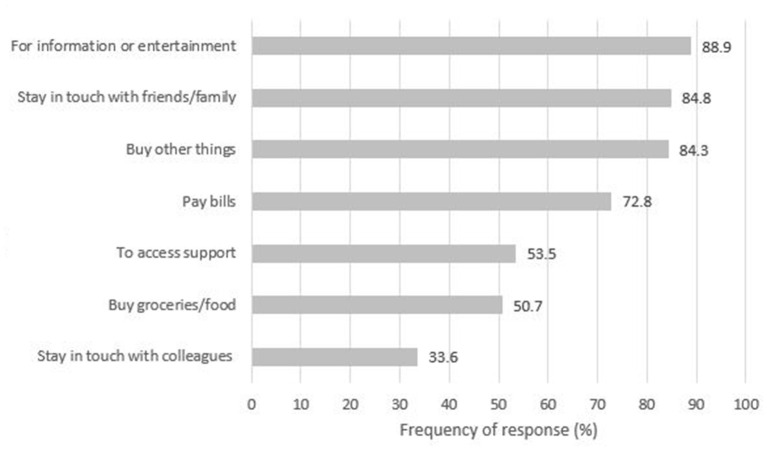
Activities performed online during the pandemic restrictions, among Internet users (limited or regular) (*N* = 217).

The most common activities that participants used the Internet for during the pandemic restrictions were to access information or entertainment (88.9%), stay in touch with friends and family (84.8%), and purchase products other than food or groceries (84.3%). The least common activity was to stay in touch with colleagues from work (33.6%) (Figure 2).

**Figure 3:**
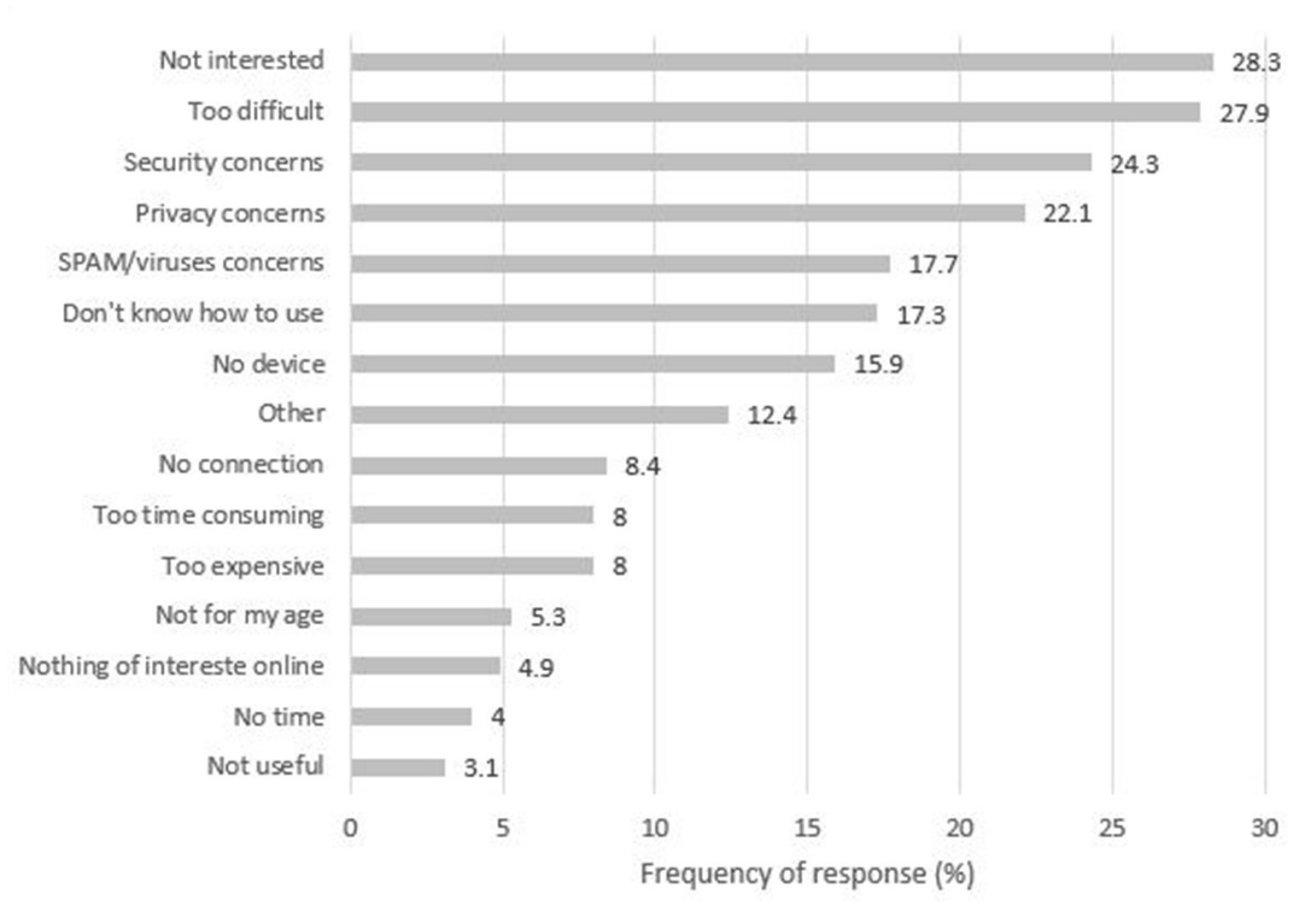
Barriers for using the Internet among limited or non-users of the Intemet (*N* = 226).

Among limited or non-users of the Internet, the most common barriers were lack of interest in using the Internet (28.3%), finding the Internet too difficult to use (27.9%), being concerned about the security of their data and information (24.3%) and being worried about their privacy (22.1%). The least reported barrier was finding the Internet not useful (3.1%) (Figure 3).

### Associations With Internet Use

In the adjusted model, younger adults (18–30 and 31–45) were five to six times more likely to have used the Internet “a lot” during the pandemic, compared to older participants (66+). Gender, ethnicity, and socioeconomic deprivation were not significantly associated with use of the Internet. Participants with a diagnosis of bipolar disorder were almost four times more likely to have used the Internet a lot during the pandemic, compared to those with a psychosis-spectrum diagnosis (Table 3).

**Table 3:** Sample characteristics associated with Internet use.

	**Using the Internet “a lot”–%** **(***N***)^a^**	**Univariate model**		**Multivariate model**	
		**OR**	**95% CI**	**Adj. OR**	**95% CI**
**Age**					
18–30	54.9 (28)	3.59^*^	1.70–7.60	6.39^**^	2.63–15.55
31–45	46.4 (45)	2.55^*^	1.34–4.87	4.76^**^	2.22–10.20
46–65	31.9 (43)	1.38	0.74–2.57	1.94	0.94–4.00
66+	25.3 (20)	1		1	
**Ethnicity**					
White	37.2 (111)	0.93	0.53–1.61	0.79	0.41–1.50
Other than White	39.3 (25)	1		1	
**Deprivation**					
Very high	31.3 (30)	0.62	0.31–1.25	0.61	0.28–1.35
High	38.8 (31)	0.86	0.42–1.76	0.91	0.40–2.07
Medium	40.9 (27)	0.94	0.45–1.97	1.15	0.50–2.64
Low	44.4 (22)	1.09	0.51–2.35	0.94	0.40–2.25
Very Low	42.3 (134)	1		1	
**Financial situation**					
Worse off	52.5 (32)	1.97 ^*^	1.13–3.44	1.73	0.89–3.34
Not worse off	35.9 (102)	1		1	
**Care setting**					
Secondary	34.2 (76)	0.68	0.44–1.05	0.84	0.50–1.39
Primary	43.5 (60)	1		1	
**Diagnosis**					
Not recorded	27.1 (13)	0.85	0.42–1.72	0.80	0.35–1.80
Other SMI	30.4 (7)	0.998	0.39–2.56	1.01	0.37–2.80
Bipolar disorder	56.7 (59)	2.99^**^	1.82–4.92	3.88^**^	2.13–7.08
Psychosis	30.5 (57)	1		1	

a *Percentages are row percentages*.

* *p < 0.05*,

** *p < 0.001*.

*Outcome variables is “using the Internet a lot” vs. “Just a little or not at all”*.

Participants who self-reported a decline in their mental health since the beginning of the pandemic were almost twice as likely to have used the Internet “a lot” during the pandemic, compared to those that did not self-report a decline (Table 4).

**Table 4:** Association of health variables with Internet use.

	**Using the internet “a lot”–%** **(***N***)^a^**	**Univariate model**		**Multivariate model**	
		**OR**	**95% CI**	**Adj. OR**	**95% CI**
**Physical health**					
Decline	45.3 (53)	1.55	0.99–2.44	1.39	0.84–2.30
No decline	34.8 (81)	1		1	
**Mental health**					
Decline	46.9 (69)	1.97^*^	1.27–3.04	1.92^*^	1.15–3.19
No decline	31.1 (64)	1		1	
**Wellbeing (Mean, SD)**	22.23 (8.46)	0.98	0.96–1.01	1.01	0.98–1.04

a *Percentages are row percentages*.

* *p < 0.05*,

** *p < 0.001*.

*Outcome variables is “using the Internet a lot” vs. “Just a little or not at all”*.

### *Post-hoc* Analysis

To further explore the association between decline in mental health and greater use of the Internet during the pandemic restrictions, we examined whether decline in mental health was associated with any of the sample characteristics that were associated with Internet use. There was a significant association with diagnosis [$x_{\left(3\right)}^{2}$ = 8.70, *P* = 0.03]. More people with bipolar disorder self-reported a mental health decline (51.9%) compared to people with psychosis (34.8%) There was no association between decline in mental health and age [$x_{\left(13\right)}^{2}$ = 2.99, *P* = 0.39].

In the light of this, we examined whether a decline in mental health was associated with greater use of the Internet after adjusting for age and diagnosis. Decline in mental health was still significantly associated with use of the Internet (*P* = 0.04, see Table 5), suggesting that decline in mental health still explained a unique portion of variance in use of the Internet after considering people's age and diagnosis.

**Table 5:** *Post-hoc* exploratory analysis: association of mental health decline with Internet use, adjusting for age, and diagnosis.

	**Using the Internet “a lot”–%** **(***N***)^a^**	**Univariate model**		**Multivariate model**	
		**OR**	**95% CI**	**Adj. OR**	**95% CI**
**Age**					
18–30	54.9 (28)	3.59^*^	1.70–7.60	5.75^**^	2.49–13.28
31–45	46.4 (45)	2.55^*^	1.34–4.87	3.23^*^	1.57–6.67
46–65	31.9 (43)	1.38	0.74–2.57	1.72	0.87–3.40
66+	25.3 (20)	1		1	
**Diagnosis**					
Not recorded	27.1 (13)	0.85	0.42–1.72	0.81	0.38–1.74
Other SMI	30.4% (7)	0.998	0.39–2.56	0.87	0.31–2.47
Bipolar disorder	56.7 (59)	2.99^**^	1.82–4.92	3.92^**^	2.24–6.87
Psychosis	30.5 (57)	1		1	
**Mental health**					
Decline	46.9 (69)	1.97^*^	1.27–3.04	1.63^*^	1.02–2.62
No decline	31.1 (64)	1			

a *Percentages are row percentages*.

* *p < 0.005*,

** *p < 0.001*.

*Outcome variables is “using the Internet a lot” vs. “Just a little or not at all”*.

## Discussion

Most people with SMI were limited or non-users of the Internet during the pandemic restrictions. Although most participants were not affected by lack of Internet or device access, an important minority did not have access to the Internet/ devices, and around half reported some knowledge gaps. Older adults with psychosis were the least likely to be regular Internet users during the pandemic.

Compared to findings among people with SMI prior to the pandemic, we found a much lower rate of non-users of the Internet [39.5 vs. 85.6% in Tobitt and Percival (24)], but also a lower rate of frequent users of the Internet [37.1 vs. 55% in Robotham et al. (23)]. Ownership of a digital device was high before the pandemic [(19, 23): 60–89% owned a computer; (20): 67.8% owned tablet and 92.8% a smartphone] and remained as such in this study (56.4% owned a computer and 79.8% owned a tablet or smartphone). It appears that the main change from pre-COVID to now is that more people are using the Internet. However, these differences should be interpreted with caution, considering sampling variations among the studies. For example, Tobitt et al. (24) recruited people with psychosis in community rehabilitation services (and therefore potentially more profoundly affected by their SMI), while Hidalgo-Mazzei et al. (20) recruited people with bipolar disorder that subscribed to an e-newsletter and Gay et al. (19) collected all their data via an online survey (thus both studies including participants potentially more confident with using online services). To minimise such bias in our study, participants had the option to complete the survey either online or offline (over the phone or with a hardcopy).

Worryingly, there seems to be a wide divide between those with SMI and the general population in terms of use of the Internet and self-reported internet skills (30). During the pandemic restrictions, 5% of the UK population was off-line (compared to 39.5% in this study), and 85% of the UK population reported feeling confident in using the Internet (compared to 48.8% reporting outstanding or good knowledge about the Internet here).

The most common barriers for using the Internet reported here (e.g., lack of interest, difficulty of the Internet, and security/privacy concerns) have also been reported by SMI studies before the pandemic [lack of knowledge, skills, or understanding: (23, 24, 31)] and in the general population during the pandemic [worry over privacy and security and finding the Internet too complicated or not interesting: (30)]. Although lack of interest might demonstrate an informed choice to not use the Internet, it might also mask deficits in skills and knowledge (32). Despite 48% of our sample residing in areas of high or very high socioeconomic deprivation, financial barriers were reported by only 8% of our sample. Financial barriers have been more prominently reported in previous studies of SMI (23). This might be explained, up to an extent, by prices in mobile data falling steeply lately (33).

Out of all the reported barriers, finding the Internet not useful was the most rarely reported (3.1%). This is positive, suggesting that most limited or non-users of the Internet recognised the benefits of engaging with the online world. This is further corroborated by the fact that almost 60% of the participants who reported a gap in their knowledge about the Internet expressed an interest in learning more about it. Futures studies should explore facilitators and barriers to taking part in a digital skills training program, as well as the most desired delivery formats and learning outcomes, to inform the development of such programs tailored to the needs of people with SMI.

Most performed activities online were accessing information and entertainment, staying in touch with friends and purchasing goods, probably due to restrictions in visiting other people or shops and spending more time in house. Least common activity was staying in touch with colleagues from work, probably since 80% of our sample was not in employment or furloughed during the pandemic.

Older people and those with a psychosis-spectrum disorders were more likely to be limited or non-users of the Internet during the pandemic. This is not surprising as older age is traditionally associated with less Internet engagement both in people with SMI (23, 24) and in the general population (11, 34). However, during the pandemic, older adults were considered at-risk for experiencing severe effects of COVID-19 and were, therefore, advised to self-isolate and not leave their premises for long periods of time. For some of them, lack of Internet engagement might have meant lack of access to essential services and support. As such, greater emphasis is needed in supporting older adults with SMI to use the Internet, as well-ensuring offline access remains available. Regarding the role of diagnosis, barriers to using the Internet related to reduced concentration, hallucinations, or paranoid ideas; (31, 35) might be more common in people with psychosis spectrum disorders than bipolar disorder. In our sample, more people with bipolar disorder (27.6%) than psychosis (12.9%) were in paid employment during the pandemic restrictions (working full or part-time and not being currently furloughed), so this might have been another reason for greater use of the Internet among people with bipolar disorder.

This study demonstrated that participants who self-reported a decline in their mental health since the beginning of the pandemic restrictions, also reported using the Internet a lot, after controlling for people's age and diagnosis. Previously, people with SMI reported being less likely to engage with digital technology when they feel less well (19). However, reports in the general UK population during the pandemic demonstrate that consumption of COVID-19 related news in social media was associated with increased depression and anxiety (36). We cannot be certain that the people who were using the internet a lot in our study were using it to access COVID-19 related news, although accessing information and entertainment was reported as the most common online activity. Furthermore, it might be that people whose mental health declined used the Internet more intensively as a coping mechanism. For example, during the COVID-19 pandemic, about half of the Internet users in the UK general population reported using the Internet to support their mental and physical health and to feel less lonely (30). This is a complex relationship that requires further investigation in terms of the online activities people were engaging with, their motivations and expectations from these activities, and the impact on their mental health. Qualitative work could be useful in further exploring these issues.

This study draws strength from its large sample size including both psychosis-spectrum and bipolar disorders and covering a range of geographic and socioeconomic areas, care settings, and ethnic backgrounds. However, the OWLS survey was kept to the shortest possible length to reduce participant burden. As a result, some of the reported variables were measured with single self-report items (e.g., participants reporting “a lot,” “a bit,” or “not at all” to “In general, have you used the Internet during the pandemic restrictions to do some of your daily activities”?) rather than more objective and fine-grained indicators (e.g., a complete break-down of activities with frequency and duration of engagement). For the same reason, we explored the common barriers found in the general population, but we did not ask our participants about barriers more specific to SMI. Despite this, this study provides important insights in use of the Internet by people with SMI during the pandemic restrictions, however, further exploration is warranted to understand more clearly some of the associations we identified.

Overall, although people with SMI may have become more digitally engaged since the pandemic began, there is still a wide gap in Internet use between people with SMI and people without SMI. However, this appears to be mainly driven by lack of skills or interest, rather than lack of Internet or device access. Digital skills among people with SMI should be further explored to understand the main areas of deficit. Digital inclusion efforts for people with SMI should focus not only on providing people with devices but also offering training and support to improve skills. It is of concern that a vulnerable sub-group (older people with psychosis) appear to be at greater risk for digital exclusion during the pandemic. Digital inclusion interventions should focus on barriers related to older age and a diagnosis of psychosis and services should try to accommodate for offline preferences in this population.

## Data Availability Statement

The raw data supporting the conclusions of this article will be made available by the authors, without undue reservation.

## Ethics Statement

The studies involving human participants were reviewed and approved by Health Research Authority Northwest—Liverpool Central Research Ethics Committee (REC reference 20/NW/0276). Written informed consent was not provided because this research involved completing a survey (online or offline) and returning the completed survey implied consent. This process was in line with the Research Ethics Committee requirements.

## Author Contributions

PS, PH, LW, SC, RW, EN, and EP contributed to the design of the survey. PS, PH, LW, and CS administered the survey to participants and collected data. PS, PH, and EP cleaned and organised the dataset. PS conducted the statistical analysis and wrote the manuscript. GJ provided guidance from a lived experience perspective. SG provided senior academic guidance. All authors contributed to the conception and design of the study, the interpretation of the findings, manuscript revision, read, and approved the submitted version.

## Funding

This study was supported by the Medical Research Council (grant reference MR/V028529) and links with the Closing the Gap cohort, which was part-funded by the Wellcome Trust (reference 204829) through the Centre for Future Health at the University of York, UK Research and Innovation (reference ES/S004459/1), and the NIHR Yorkshire and Humberside Applied Research Collaboration.

## Author Disclaimer

Any views expressed here are those of the project investigators and do not necessarily represent the views of the Medical Research Council, Wellcome Trust, UK Research and Innovation, National Institute for Health Research or the Department of Health and Social Care.

## Conflict of Interest

The authors declare that the research was conducted in the absence of any commercial or financial relationships that could be construed as a potential conflict of interest.

## Publisher's Note

All claims expressed in this article are solely those of the authors and do not necessarily represent those of their affiliated organizations, or those of the publisher, the editors and the reviewers. Any product that may be evaluated in this article, or claim that may be made by its manufacturer, is not guaranteed or endorsed by the publisher.
